# Supplementary material for: On the Origin of Raman Activity in Anatase TiO2 (Nano)Materials: An Ab Initio Investigation of Surface and Size Effects
Source: Nanomaterials (Basel). 2023 Jun 14;13(12):1856. doi: 10.3390/nano13121856 (PMC10301693; doi:10.3390/nano13121856)
Supplement: Supplementary file 1 [file nanomaterials-13-01856-s001.zip › nanomaterials-2426500-supplementary.pdf]

On the origin of Raman activity in anatase TiO_2 (nano)materials : an *ab initio* investigation of surface and size effects

B. Taudul[1], F. Tielens[2], M. Calatayud[1*]

[1] Sorbonne Université, CNRS, Laboratoire de Chimie Théorique, LCT, 4 Place Jussieu, F-75005 Paris, France

[2] Research Group of General Chemistry (ALGC) – Materials Modelling Group, Vrije Universiteit Brussel, Pleinlaan 2, 1050 Brussel, Belgium

Supplementary information

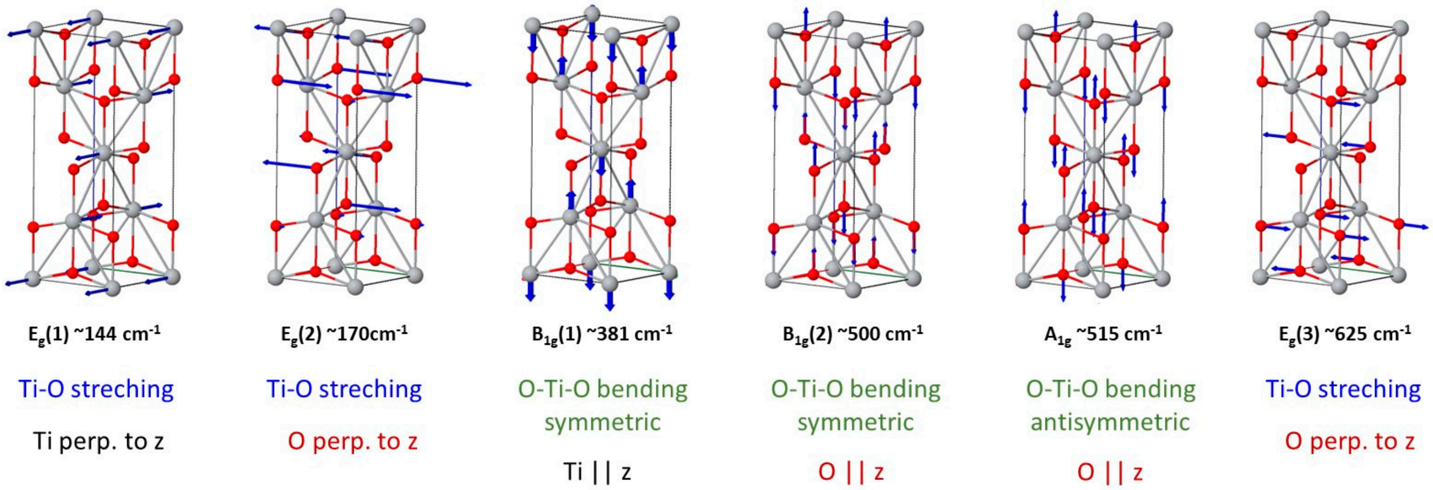

Figure S1. Raman active vibrational modes of bulk anatase. Picture made with CRYSPLOT.

Table S1. Geometrical parameters of anatase (101), (100) and (001) surface models and associated surface energy for each slab.

Termination	*a* [A]	b [A]	α=β	γ	Nr of ML	Nr of atoms	Thick. [A]	Thick. [nm]	Surface energy [J/m²]
**(101)**	3.784	5.436	90	110.4	6	18	9.3	0.9	0.640
					8	24	12.8	1.3	0.659
					10	30	16.3	1.6	0.672
					12	36	19.8	2	0.680
					14	42	23.3	2.3	0.687
					16	48	26.8	2.7	0.694
					18	54	30.4	3	0.698
					20	60	33.9	3.4	0.703

**(100)**	3.784	3.784	90	90	5	30	8.1	0.8	0.838
					6	36	9.8	1	0.763
					7	42	11.8	1.2	0.820
					8	48	13.7	1.4	0.787
					9	54	15.6	1.6	0.809
					10	60	17.5	1.7	0.796
					11	66	19.4	1.9	0.807
					12	72	21.2	2.1	0.803
					13	78	23.1	2.3	0.808
					14	84	25	2.5	0.807
**(001)**	3.784	3.784	90	90	6	18	12.6	1.3	1.351
					8	24	17.4	1.7	1.352
					10	30	22.1	2.2	1.353
					12	36	26.8	2.7	1.356
					14	42	31.6	3.2	1.359
					16	48	36.3	3.6	1.364
					20	60	45.8	4.6	1.376
					24	72	55.3	5.5	1.395

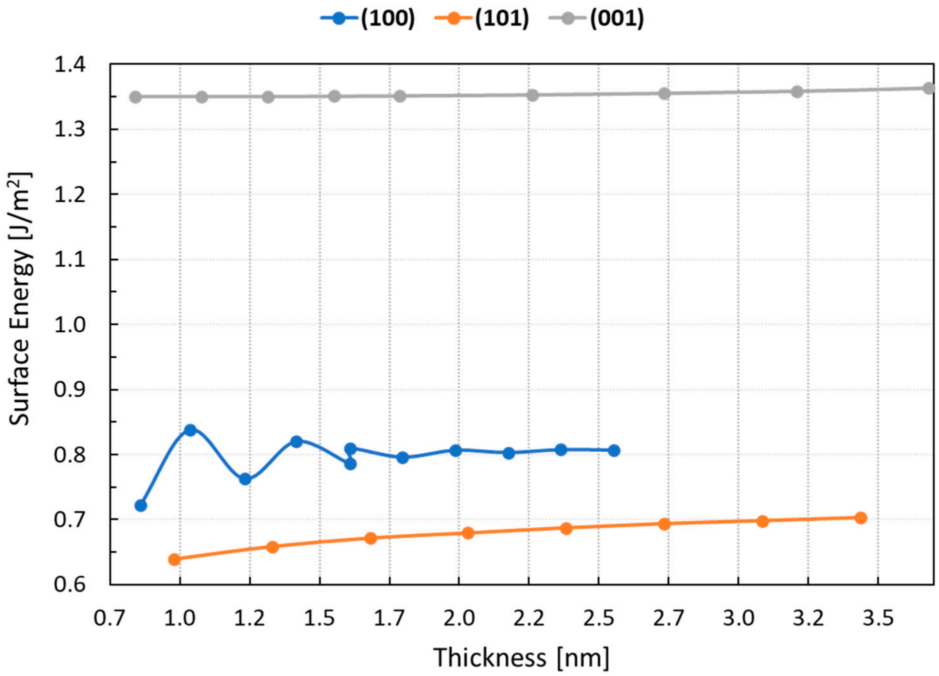

Figure S2. Evolution of the surface energy with slab thickness for anatase (101), (100) and (001) terminations.

Table S2. Ti-O bond lengths in Angstroms for **(101)** slabs with different number of layers. Slabs are symmetric so only half of the layers has to be described. Colors in the table correspond to colors of different oxygen types present in (101) slabs.

	6ML	8ML	10ML	12ML	14ML	16ML	18ML	20ML
O1-Ti1	1.822	1.821	1.820	1.819	1.819	1.819	1.819	1.819
O1-Ti2	1.836	1.841	1.844	1.845	1.846	1.847	1.847	1.847
O2-Ti1	1.968	1.968	1.968	1.968	1.969	1.969	1.969	1.969
O2-Ti2	1.977	1.978	1.979	1.980	1.980	1.980	1.980	1.980
O3-Ti1	2.018	2.022	2.025	2.026	2.026	2.027	2.027	2.027
O3-Ti2	1.927	1.927	1.927	1.927	1.927	1.927	1.927	1.927
O4-Ti1	1.780	1.779	1.779	1.779	1.780	1.780	1.780	1.780
O4-Ti2	2.057	2.063	2.067	2.068	2.069	2.069	2.069	2.069
O4-Ti3	2.024	2.004	1.995	1.990	1.988	1.987	1.986	1.986
O5-Ti2	2.085	2.080	2.078	2.076	2.076	2.076	2.075	2.075
O5-Ti3	1.960	1.953	1.948	1.945	1.943	1.942	1.941	1.941
O5-Ti4	1.866	1.890	1.904	1.911	1.916	1.919	1.920	1.920
O6-Ti3	1.929	1.931	1.932	1.933	1.933	1.933	1.933	1.934
O6-Ti4	1.979	1.972	1.969	1.968	1.967	1.967	1.966	1.966
O7-Ti4	1.929	1.928	1.927	1.927	1.927	1.927	1.927	1.926
O7-Ti3	1.979	1.987	1.993	1.996	1.998	1.999	1.999	1.999
O8-Ti3	-	1.861	1.862	1.862	1.863	1.863	1.864	1.864
O8-Ti4	-	1.971	1.980	1.984	1.987	1.988	1.989	1.989
O8-Ti5	-	2.014	1.986	1.972	1.966	1.962	1.961	1.960
O9-Ti4	-	2.014	2.003	1.998	1.994	1.993	1.992	1.992
O9-Ti5	-	1.971	1.968	1.966	1.964	1.963	1.963	1.962
O9-Ti6	-	1.861	1.888	1.903	1.912	1.916	1.918	1.919
O10-Ti5	-	-	1.930	1.931	1.931	1.931	1.932	1.932
O10-Ti6	-	-	1.979	1.976	1.974	1.973	1.973	1.973
O11-Ti5	-	-	1.979	1.983	1.986	1.987	1.987	1.988
O11-Ti6	-	-	1.930	1.929	1.929	1.929	1.929	1.929
O12-Ti5	-	-	-	1.891	1.892	1.893	1.894	1.894
O12-Ti6	-	-	-	1.976	1.980	1.983	1.983	1.984
O12-Ti7	-	-	-	1.974	1.960	1.953	1.950	1.949
O13-Ti6	-	-	-	1.974	1.968	1.964	1.963	1.962
O13-Ti7	-	-	-	1.976	1.974	1.973	1.972	1.971
O13-Ti8	-	-	-	1.891	1.907	1.915	1.920	1.922
O14-Ti7	-	-	-	1.929	1.930	1.931	1.931	1.931
O14-Ti8	-	-	-	1.983	1.980	1.979	1.977	1.977
O15-Ti7	-	-	-	1.976	1.980	1.982	1.983	1.984
O15-Ti8	-	-	-	1.931	1.930	1.930	1.930	1.930
O16-Ti7	-	-	-	-	-	1.908	1.909	1.910
O16-Ti8	-	-	-	-	-	1.978	1.981	1.982
O16-Ti9	-	-	-	-	-	1.953	1.946	1.943
O17-Ti8	-	-	-	-	-	1.953	1.950	1.949

O17-Ti9	-	-	-	-	-	1.978	1.977	1.976
O17-Ti10	-	-	-	-	-	1.908	1.917	1.922
O18-Ti9	-	-	-	-	-	-	1.930	1.931
O18-Ti10	-	-	-	-	-	-	1.980	1.980
O19-Ti9	-	-	-	-	-	-	1.980	1.981
O19-Ti10	-	-	-	-	-	-	1.930	1.930

Table S3. Calculated Raman frequencies and total Raman intensities (raw and with temperature/laser correction) for **(101) with 12ML**. The modes in bold are visualized in Fig. S3.

Freq. (cm^{-1})	Symm.	Int.	Int. + temp.
29.18	(Bg)	2.03	116.8
59.01	(Ag)	7.73	115.59
76.75	(Bg)	2.88	26.44
82.26	(Ag)	1.34	10.8
97.44	(Bg)	0.86	5.08
123.18	(Ag)	0.18	0.7
**144.52**	(Bg)	154.74	457.14
**145.65**	(Bg)	343.04	1000
**157.66**	(Bg)	115.27	293.39
159.98	(Ag)	23.03	57.18
172.46	(Ag)	0.44	0.96
184.59	(Ag)	30.25	59.04
184.82	(Bg)	68.77	133.96
197.74	(Ag)	6.6	11.5
199.92	(Ag)	3.13	5.36
207.25	(Bg)	23.01	37.13
242.37	(Ag)	29.37	36.86
245.29	(Bg)	0.3	0.37
246.97	(Ag)	1.06	1.29
256.88	(Bg)	0.26	0.29
277.08	(Bg)	0.36	0.37
279.30	(Ag)	0.77	0.77
284.11	(Ag)	1.29	1.27
292.97	(Bg)	3.94	3.69
297.28	(Ag)	13.75	12.58
315.57	(Ag)	26.42	22.09
352.07	(Ag)	10.03	7.13
381.94	(Ag)	28.28	17.88
**397.96**	(Ag)	186.21	111.03
402.31	(Ag)	65.72	38.59
403.55	(Bg)	0.79	0.46
410.76	(Bg)	0.69	0.39
419.28	(Ag)	3.74	2.07
429.57	(Bg)	0.24	0.13

440.79	(Ag)	10.83	5.6
450.07	(Ag)	20.47	10.28
489.33	(Ag)	22.82	10.22
**504.63**	(Ag)	89.36	38.38
**504.97**	(Ag)	18.42	7.9
**506.92**	(Ag)	154.77	66.07
**515.58**	(Ag)	235.05	98.08
520.90	(Ag)	10.91	4.49
532.91	(Ag)	12.4	4.95
590.22	(Ag)	59.62	20.8
**601.87**	(Bg)	422.6	143.74
**620.08**	(Bg)	163.32	53.44
**624.77**	(Bg)	1000	324.03
665.27	(Ag)	4.4	1.31
710.35	(Ag)	0.39	0.11
**731.68**	(Ag)	102.8	27.21
763.54	(Ag)	0.15	0.04
828.31	(Ag)	1.2	0.27
893.45	(Ag)	17.81	3.66

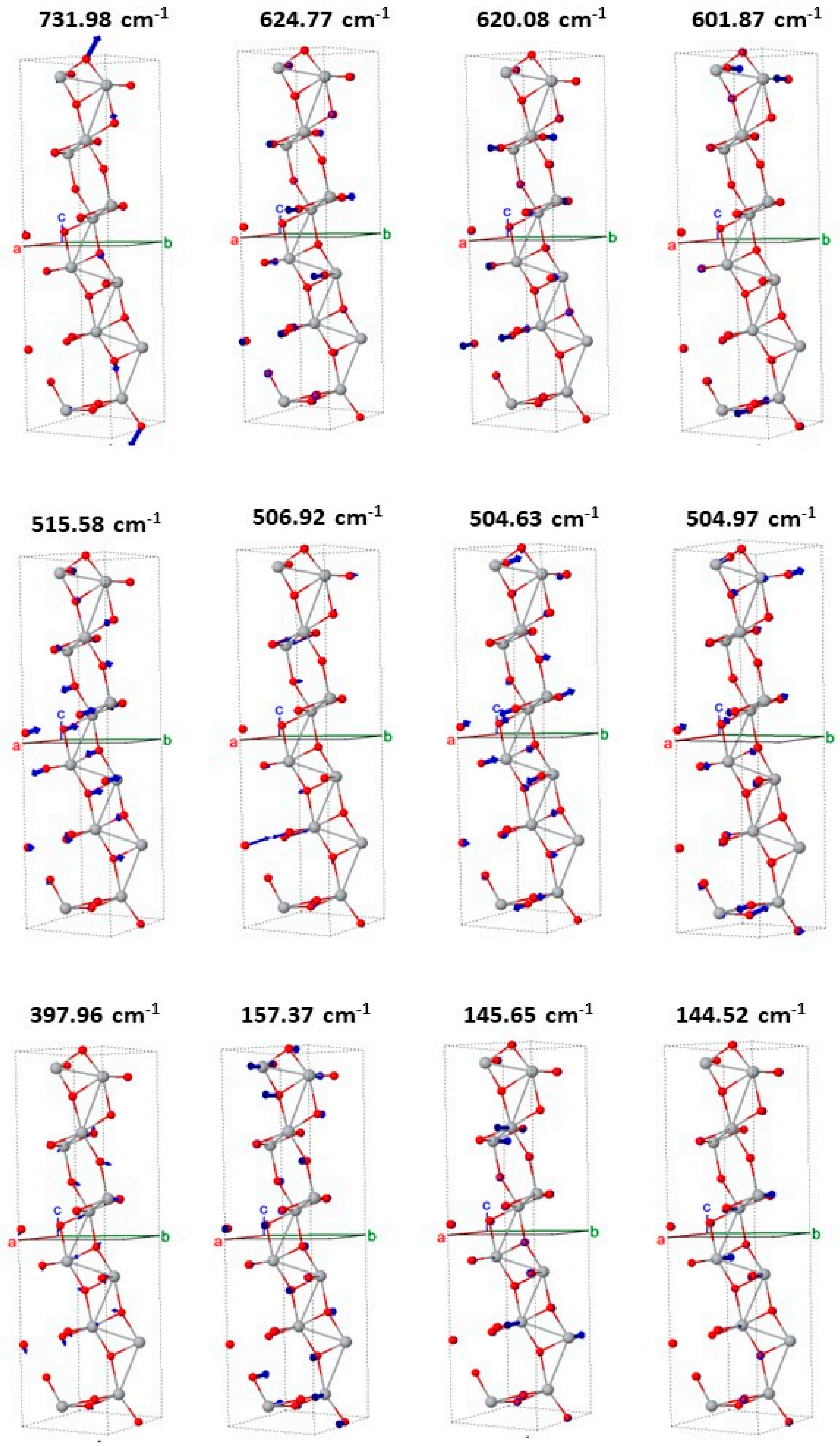

Figure S3. Visualization of the chosen modes for (101) 12ML structure

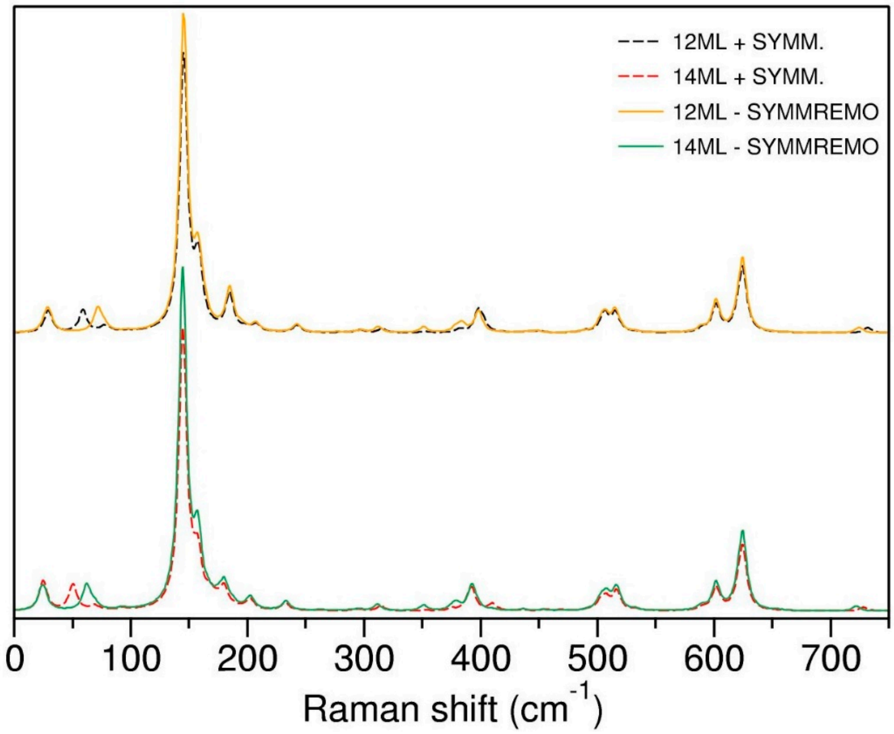

Figure S4. Comparison of Raman spectra for 12 and 14ML slabs of (101) termination obtained with ('SYMM.') and without ('SYMMREMO.') symmetry constraints.

Table S4. Ti-O bond lengths in Angstroms for **(001)** slabs with different number of layers. Parameters correspond to (001) structures with symmetric Ti-O bonds in the surface layer. Colors in the table correspond to colors of different oxygen types present in (001) slabs.

	**6ML**	**8ML**	**10ML**	**12ML**	**14ML**	**16ML**	**24ML**
O1-Ti1	1.949	1.949	1.949	1.949	1.949	1.949	1.950
O2-Ti1	1.922	1.922	1.922	1.922	1.922	1.922	1.922
O2-Ti2	1.957	1.957	1.957	1.957	1.957	1.957	1.959
O3-Ti1	1.913	1.913	1.913	1.913	1.913	1.914	1.915
O3-Ti2	1.930	1.930	1.930	1.930	1.930	1.930	1.930
O4-Ti2	1.933	1.933	1.933	1.933	1.933	1.933	1.933
O4-Ti3	1.977	1.977	1.977	1.977	1.977	1.977	1.979
O5-Ti2	1.983	1.983	1.983	1.983	1.983	1.983	1.984
O5-Ti3	1.932	1.932	1.932	1.932	1.932	1.932	1.932
O6-Ti3	1.931	1.931	1.931	1.931	1.931	1.931	1.931
O6-Ti4	1.983	1.981	1.982	1.982	1.982	1.982	1.983
O7-Ti3	1.983	1.982	1.982	1.982	1.982	1.982	1.983
O7-Ti4	1.931	1.931	1.931	1.931	1.931	1.931	1.931

O8-Ti4	1.932	1.931	1.931	1.931	1.931	1.931	1.931
O8-Ti5	1.983	1.980	1.980	1.980	1.980	1.981	1.981
O9-Ti4	1.977	1.980	1.980	1.980	1.980	1.980	1.981
O9-Ti5	1.933	1.931	1.931	1.931	1.931	1.931	1.931
O10-Ti5	-	1.931	1.931	1.931	1.931	1.931	1.931
O10-Ti6	-	1.982	1.980	1.980	1.980	1.980	1.981
O11-Ti5	-	1.981	1.980	1.980	1.980	1.980	1.981
O11-Ti6	-	1.931	1.931	1.931	1.931	1.931	1.931
O12-Ti6	-	-	1.931	1.931	1.931	1.931	1.931
O12-Ti7	-	-	1.980	1.980	1.980	1.980	1.981
O13-Ti6	-	-	1.980	1.980	1.980	1.980	1.981
O13-Ti7	-	-	1.931	1.931	1.931	1.931	1.931
O14-TI7	-	-	-	-	1.931	1.931	1.931
O14-Ti8	-	-	-	-	1.980	1.980	1.981
O15-Ti7	-	-	-	-	1.980	1.980	1.981
O15-Ti8	-	-	-	-	1.931	1.931	1.931
O16-Ti8	-	-	-	-	-	1.931	1.931
O16-Ti9	-	-	-	-	-	1.980	1.981
O17-Ti8	-	-	-	-	-	1.980	1.981
O17-Ti9	-	-	-	-	-	1.931	1.931
O18-Ti9	-	-	-	-	-	1.931	1.931
O18-Ti10	-	-	-	-	-	1.980	1.981
O19-Ti9	-	-	-	-	-	1.980	1.981
O19-Ti10	-	-	-	-	-	1.931	1.931

Table S5. Calculated Raman frequencies and total Raman intensities (raw and with temperature/laser correction) for **(001) with 12ML**. The modes in bold are visualized in Fig. S5.

Freq.	Symm.	Int.	Int.+T
19.16	(B2g)	0.15	48.72
19.53	(B3g)	0.15	45.53
39.57	(Ag)	4.34	343.68
50.57	(B3g)	0.07	3.53
53.57	(B2g)	0.07	3.14
66.19	(B3g)	0	0.08
83.18	(B2g)	0.08	1.63
121.22	(Ag)	2.2	21.93
**144.85**	(B3g)	17.67	128.94
**144.94**	(B2g)	20.86	152.04
149.67	(B2g)	1.7	11.7
151.33	(B3g)	1.25	8.49

155.94	(B3g)	0.97	6.25
157.35	(B2g)	0.11	0.68
162.14	(B2g)	0	0
162.36	(B3g)	0.06	0.33
168.63	(B2g)	0.06	0.32
168.68	(B3g)	0.04	0.24
192.65	(B3g)	2.16	9.74
196.39	(Ag)	1.2	5.24
212.66	(B2g)	1.02	3.91
242.80	(B3g)	0	0.01
244.32	(B2g)	0.18	0.54
261.03	(B3g)	0	0
262.46	(B2g)	0.07	0.2
264.82	(Ag)	2.82	7.62
295.60	(B3g)	2.18	4.98
326.09	(B2g)	7.37	14.55
326.40	(Ag)	27.59	54.4
**373.26**	(Ag)	601.02	973.8
411.32	(B3g)	0	0
415.95	(B2g)	0.05	0.06
**420.72**	(Ag)	372.65	509.41
424.72	(B3g)	0	0
427.31	(B2g)	0.03	0.04
476.23	(B3g)	0.48	0.55
487.66	(Ag)	1.24	1.39
493.05	(Ag)	2.83	3.11
**499.38**	(Ag)	240.28	259.51
**504.88**	(Ag)	136.9	145.69
**514.85**	(Ag)	661.53	685.73
**528.87**	(Ag)	1000	1000
569.79	(Ag)	6.86	6.22
616.47	(Ag)	1.57	1.28
**625.02**	(B3g)	42.39	34.04
**625.36**	(B2g)	37.24	29.89
**625.70**	(B3g)	0.59	0.48
**626.26**	(B2g)	2.47	1.98
**627.43**	(B2g)	13.7	10.94
**650.62**	(B3g)	15.25	11.62
661.58	(Ag)	0.02	0.02
696.51	(Ag)	2.28	1.59
715.26	(Ag)	3.01	2.03

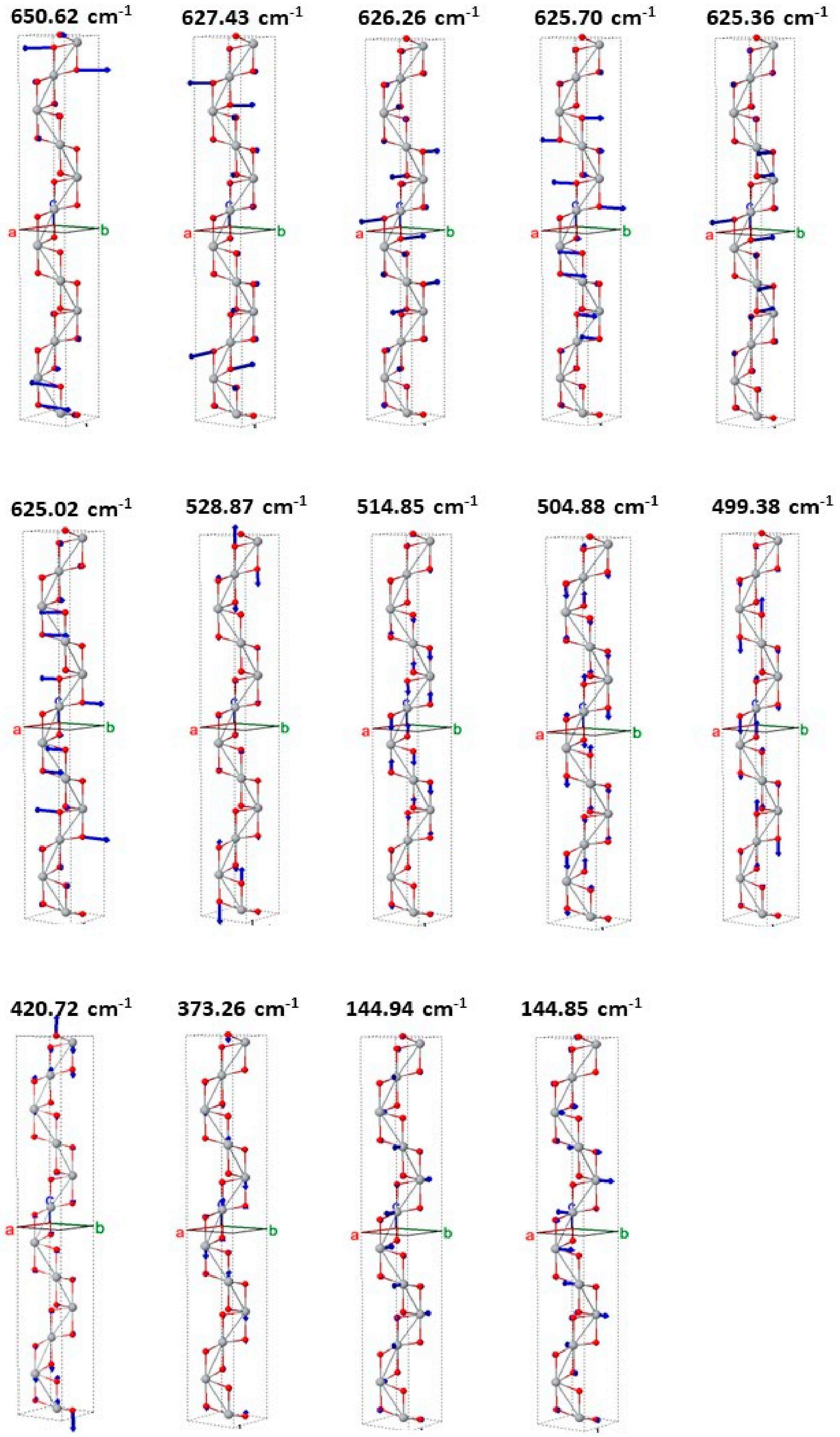

Figure S5. Visualization of the chosen modes for 12ML (001) structure. Figures were prepared using CRYSPLOT tool.

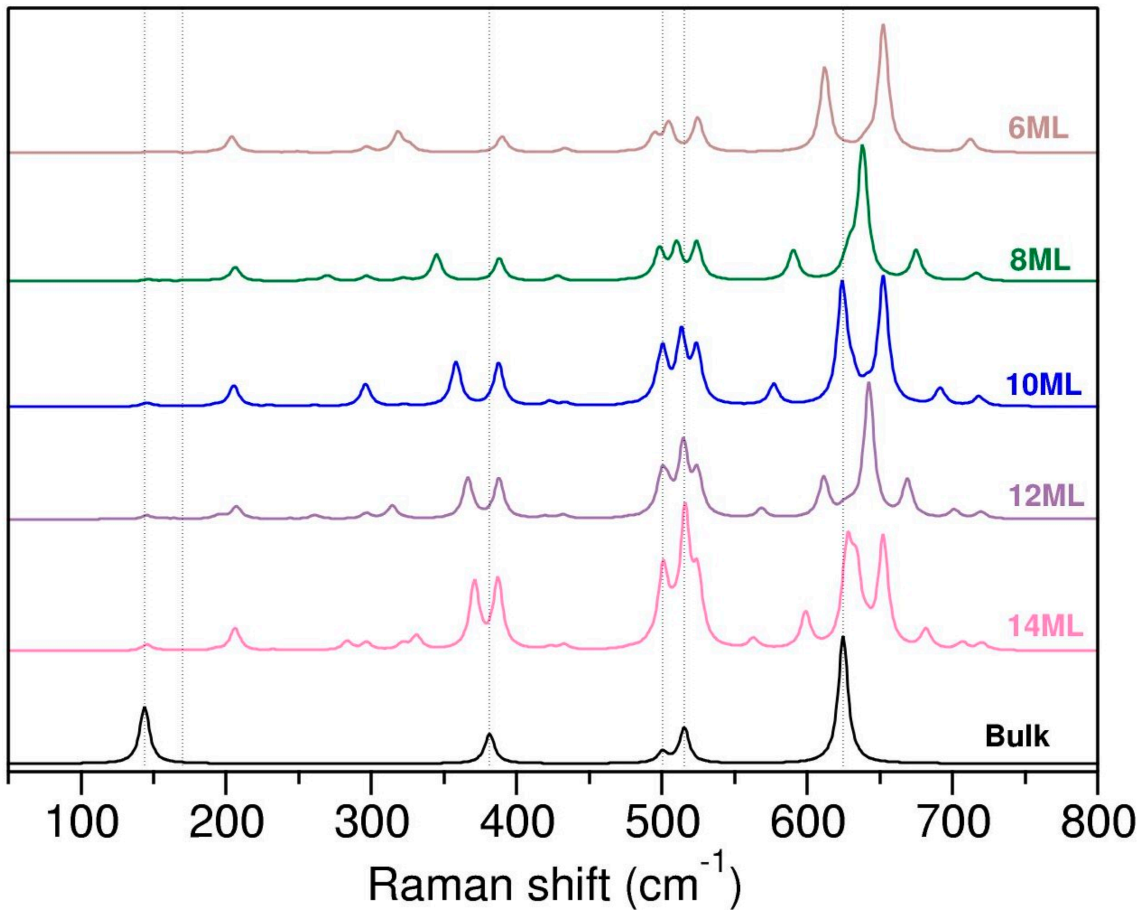

Figure S6. Raman spectra for (001) slabs with non-symmetric Ti-O bonds on the surface (structures optimized without symmetry constrains) and without temperature/laser correction for intensities.

Table S6. Ti-O bond lengths in Angstroms for selected "non-symmetric" **(001)**. Only the top most distances are affected, the other bonds are as for the "symmetric" (001) slabs.

	6ML	8ML	**10ML**	**12ML**	**14ML**
O1-Ti1	1.748	1.748	1.748	1.748	1.821
O1-Ti1	2.189	2.189	2.189	2.189	2.078
O2-Ti1	1.936	1.936	1.936	1.936	1.926
O2-Ti2	1.958	1.958	1.958	1.958	1.957
O3-Ti1	1.915	1.915	1.915	1.915	1.918
O3-Ti2	1.933	1.933	1.933	1.933	1.930
O4-Ti2	1.965	1.965	1.965	1.965	1.933
O4-Ti3	1.977	1.977	1.977	1.978	1.977
O5-Ti2	1.985	1.985	1.985	1.985	1.983
O5-Ti3	1.931	1.933	1.933	1.930	1.932

Table S7. Ti-O bond lengths in Angstroms for **(100)** slabs with different number of layers.

	6ML	8ML	10ML	12ML	14ML		5ML	7ML	9ML	11ML	13ML
O1-Ti1	2.044	2.053	2.056	2.057	2.058	O1-Ti1	2.080	2.067	2.061	2.059	2.059
O1-Ti2	1.961	1.962	1.962	1.962	1.962	O1-Ti2	1.965	1.963	1.963	1.963	1.963
O2-Ti1	1.961	1.962	1.962	1.962	1.962	O2-Ti1	1.965	1.963	1.963	1.963	1.963
O2-Ti2	2.044	2.053	2.056	2.057	2.058	O2-Ti2	2.080	2.067	2.061	2.059	2.059
O3-Ti1	1.821	1.819	1.818	1.817	1.817	O3-Ti1	1.813	1.813	1.815	1.816	1.816
O3-Ti4	1.839	1.845	1.848	1.849	1.850	O3-Ti4	1.866	1.859	1.854	1.851	1.850
O4-Ti2	1.821	1.819	1.818	1.817	1.817	O4-Ti2	1.813	1.813	1.815	1.816	1.816
O4-Ti3	1.839	1.845	1.848	1.849	1.850	O4-Ti3	1.866	1.859	1.854	1.851	1.850
O5-Ti3	1.981	1.981	1.980	1.979	1.979	O5-Ti3	1.978	1.976	1.977	1.978	1.979
O5-Ti4	1.935	1.935	1.934	1.934	1.934	O5-Ti4	1.935	1.933	1.934	1.934	1.934
O6-Ti3	1.935	1.935	1.934	1.934	1.934	O6-Ti3	1.935	1.933	1.934	1.934	1.934
O6-Ti4	1.981	1.981	1.980	1.979	1.979	O6-Ti4	1.978	1.976	1.977	1.978	1.979
O7-Ti2	1.790	1.791	1.793	1.793	1.793	O7-Ti2	1.795	1.796	1.795	1.794	1.794
O7-Ti3	2.090	2.085	2.086	2.087	2.087	O7-Ti3	2.105	2.090	2.091	2.089	2.088
O7-Ti6	1.996	1.980	1.972	1.968	1.967	O7-Ti6	1.922	1.946	1.957	1.962	1.965
O9-Ti5	1.977	1.981	1.985	1.987	1.988	O9-Ti5	2.011	1.998	1.993	1.990	1.989
O9-Ti6	1.929	1.929	1.929	1.930	1.930	O9-Ti6	1.928	1.931	1.930	1.930	1.930
O11-Ti4	2.035	2.039	2.037	2.036	2.035	O11-Ti4	2.029	2.030	2.030	2.033	2.034
O11-Ti5	1.963	1.955	1.954	1.952	1.951	O11-Ti5	1.902	1.942	1.945	1.947	1.949
O11-Ti8	1.875	1.900	1.912	1.918	1.920	O11-Ti8	2.029	1.961	1.939	1.929	1.925
O13-Ti7	-	1.979	1.978	1.977	1.977	O13-Ti7	-	1.976	1.973	1.975	1.976
O13-Ti8	-	1.932	1.931	1.931	1.930	O13-Ti8	-	1.931	1.929	1.930	1.930
O15-Ti6	-	1.876	1.878	1.880	1.881	O15-Ti6	-	1.890	1.888	1.885	1.883
O15-Ti7	-	1.988	1.984	1.984	1.985	O15-Ti7	-	1.988	1.985	1.987	1.986
O15-Ti10	-	1.975	1.956	1.946	1.942	O15-Ti10	-	1.890	1.915	1.929	1.935
O17-Ti9	-	-	1.980	1.982	1.984	O17-Ti9	-	-	1.991	1.988	1.986
O17-Ti10	-	-	1.931	1.932	1.932	O17-Ti10	-	-	1.934	1.933	1.933
O19-Ti8	-	-	1.977	1.973	1.971	O19-Ti8	-	-	1.961	1.964	1.967
O19-Ti9	-	-	1.983	1.984	1.982	O19-Ti9	-	-	1.978	1.977	1.978
O19-Ti12	-	-	1.900	1.912	1.918	O19-Ti12	-	-	1.961	1.940	1.930
O21-Ti11	-	-	-	1.979	1.977	O21-Ti11	-	-	-	1.973	1.975
O21-Ti12	-	-	-	1.930	1.930	O21-Ti12	-	-	-	1.929	1.929
O23-Ti10	-	-	-	1.902	1.905	O23-Ti10	-	-	-	1.914	1.910
O23-Ti11	-	-	-	1.980	1.980	O23-Ti11	-	-	-	1.982	1.984
O23-Ti14	-	-	-	1.956	1.946	O23-Ti14	-	-	-	1.914	1.928
O26-Ti13	-	-	-	-	1.931	O26-Ti13	-	-	-	-	1.932
O26-Ti14	-	-	-	-	1.981	O26-Ti14	-	-	-	-	1.987
O27-Ti12	-	-	-	-	1.952	O27-Ti12	-	-	-	-	1.943
O27-Ti13	-	-	-	-	1.980	O27-Ti13	-	-	-	-	1.975
O27-Ti16	-	-	-	-	1.915	O27-Ti16	-	-	-	-	1.943

Table S8. Calculated Raman frequencies and total Raman intensities (raw and with temperature/laser correction) for (100) with 11 and 12ML.

11ML				12ML			
Freq. (cm-1)	Symm.	Int.	Int. + temp.	Freq. (cm-1)	Symm.	Int.	Int. + temp.
27.36	(B3g)	0.18	8	24.91	(B3g)	0.1	7.26
29.89	(B1g)	0.04	1.35	27.47	(B1g)	0.02	1.23
53.75	(Ag)	35.82	444.06	51.07	(Ag)	20.52	374.06
68.62	(B3g)	0.08	0.63	71.46	(B1g)	0	0
75.03	(B1g)	0.01	0.05	72.44	(B1g)	0.05	0.45
80.29	(B1g)	0.07	0.39	81.75	(B3g)	0.11	0.81
88.73	(B1g)	0.04	0.2	83.65	(B1g)	0	0
88.80	(B3g)	0.06	0.31	85.90	(B3g)	0.01	0.05
102.59	(B1g)	0.11	0.42	94.10	(B1g)	0.03	0.19
113.37	(B3g)	1.64	5.17	103.06	(B1g)	0.05	0.26
127.35	(B3g)	0.05	0.13	122.02	(B3g)	0.3	1.1
136.52	(B2g)	64.83	147.04	134.46	(B3g)	0.86	2.67
140.29	(B2g)	462.28	1000	139.33	(B2g)	72.85	212.88
150.08	(B3g)	6.79	13.07	143.69	(B2g)	360.95	1000
156.62	(B2g)	20.05	35.9	150.22	(B2g)	0.49	1.25
159.56	(B2g)	125.24	217.24	158.30	(B2g)	42.47	99.67
163.04	(B2g)	32.98	55.15	159.23	(B3g)	4.21	9.79
167.58	(Ag)	10.57	16.87	159.47	(Ag)	15.94	36.94
168.25	(B3g)	0.02	0.03	163.41	(B2g)	47.5	105.64
168.60	(B2g)	96.36	152.24	164.36	(B1g)	0.36	0.79
173.89	(B3g)	0.02	0.03	168.57	(B2g)	63.8	134.6
182.50	(B1g)	0.47	0.65	171.35	(B3g)	0.26	0.53
184.47	(B3g)	1.25	1.7	177.22	(B3g)	2.31	4.49
187.01	(B2g)	76.56	101.72	179.36	(B3g)	0.58	1.11
187.82	(B3g)	2.83	3.73	179.63	(Ag)	2.09	3.96
189.03	(Ag)	1.39	1.82	185.06	(B2g)	48.96	88.37
193.66	(B3g)	0.68	0.85	186.53	(B3g)	0.23	0.41
195.00	(B3g)	0.13	0.16	188.31	(Ag)	0.7	1.23
197.96	(Ag)	1.34	1.62	192.75	(B3g)	0.32	0.54
215.46	(B2g)	19.32	20.36	197.01	(B3g)	0.01	0.01
220.27	(B3g)	0.04	0.04	198.93	(B1g)	0.11	0.18
223.30	(Ag)	0.87	0.86	201.56	(Ag)	0.81	1.27
224.55	(B1g)	0	0	213.50	(B2g)	16.91	24.13
236.24	(Ag)	12.64	11.49	220.21	(B3g)	0	0
246.04	(B3g)	0.05	0.05	228.15	(Ag)	0.87	1.12
250.03	(B1g)	0	0	238.59	(Ag)	0.45	0.53
255.07	(B2g)	0.14	0.11	241.37	(B3g)	0.01	0.02
266.43	(Ag)	7.62	5.74	242.52	(B1g)	0.01	0.01
269.67	(B2g)	1.01	0.75	247.43	(B2g)	0.01	0.01
270.61	(B1g)	0.02	0.02	248.07	(B1g)	0	0
277.79	(B2g)	0.46	0.33	259.79	(B3g)	0.15	0.15
284.56	(Ag	2.45	1.66	268.25	(B1g)	0	0
287.87	(B1g)	0.56	0.37	272.08	(Ag	7.98	7.76
290.42	(B3g)	0.05	0.03	273.98	(B2g)	0.9	0.86
298.59	(B2g)	0.47	0.3	278.64	(B2g)	0.04	0.03
298.75	(Ag	0.26	0.17	286.56	(B3g)	0.01	0.01
304.72	(B3g)	0	0	288.88	(B1g)	0.35	0.31
316.71	(Ag)	0.52	0.3	289.03	(Ag)	1.77	1.57
321.30	(B3g)	0.19	0.11	291.18	(Ag)	1.17	1.03
323.61	(Ag)	15.91	8.91	297.32	(Ag)	0.12	0.1
369.50	(B3g)	3.13	1.44	325.17	(B3g)	0.08	0.06
373.36	(B1g)	10.29	4.67	329.86	(Ag)	12.88	9.36

394.26	(B1g)	0	0	359.82	(Ag)	41.09	26.29
402.18	(Ag)	40.03	16.36	368.81	(B3g)	1.75	1.08
406.72	(B1g)	0.16	0.06	375.51	(B1g)	6.81	4.1
411.71	(B1g)	0	0	376.15	(B3g)	0.36	0.22
422.97	(Ag)	18.41	7.01	388.17	(Ag)	150.3	86.22
425.88	(B3g)	2.01	0.76	400.70	(B1g)	0.02	0.01
426.63	(B1g)	0.04	0.01	404.56	(Ag)	21.7	11.74
433.62	(B3g)	0.23	0.09	405.65	(B1g)	0	0
443.89	(B3g)	0.17	0.06	410.48	(B1g)	0.17	0.09
461.75	(Ag	22.77	7.67	413.94	(B1g)	0.01	0
470.81	(B3g)	0.14	0.05	428.42	(B1g)	0.03	0.01
474.32	(Ag)	10.01	3.25	428.46	(B3g)	0.08	0.04
488.58	(Ag)	2.99	0.93	429.64	(Ag)	11.44	5.69
490.17	(Ag)	24.37	7.57	439.26	(B3g)	0.71	0.34
491.96	(B3g)	0.61	0.19	452.85	(B3g)	0.04	0.02
499.24	(Ag)	79.96	24.24	462.41	(Ag)	15.85	7.12
500.22	(Ag)	144.98	43.83	463.14	(B3g)	0.58	0.26
504.68	(B3g)	3.71	1.11	470.71	(Ag)	8.57	3.76
506.97	(Ag)	30.11	8.94	484.50	(B3g)	0	0
516.77	(Ag)	363.37	105.14	489.33	(Ag)	4.29	1.78
522.86	(Ag)	59.27	16.88	492.03	(Ag)	1.19	0.49
530.64	(Ag)	4.54	1.27	493.29	(B3g)	0.02	0.01
567.25	(Ag)	52.55	13.44	497.44	(Ag)	13.09	5.32
581.50	(B3g)	12.68	3.14	498.95	(Ag)	182.63	73.95
589.52	(B2g)	233.16	56.68	505.50	(Ag)	18.3	7.28
617.76	(B2g)	40.63	9.29	510.55	(B3g)	2.84	1.12
620.28	(B2g)	4.73	1.08	514.83	(Ag)	105.17	40.83
622.09	(B2g)	1000	226.64	517.50	(Ag)	189.18	72.93
623.37	(B2g)	210.65	47.61	527.36	(Ag)	6.15	2.31
627.40	(B2g)	588.05	131.82	546.88	(Ag)	9.13	3.27
654.01	(B3g)	0.47	0.1	582.63	(B3g)	9.49	3.13
668.99	(B3g)	11.32	2.34	591.04	(B2g)	157.58	50.96
681.78	(Ag)	26.69	5.38	611.50	(Ag)	45.3	14.02
690.58	(B3g)	0.51	0.1	618.66	(B2g)	18.34	5.59
706.96	(B3g)	0.03	0.01	620.19	(B2g)	17.11	5.2
707.69	(Ag)	18.65	3.58	621.98	(B2g)	181.54	54.94
759.59	(Ag)	47.96	8.42	624.27	(B2g)	138.73	41.78
766.86	(B3g)	0.26	0.05	625.24	(B2g)	1000	300.57
776.50	(Ag)	0.41	0.07	641.35	(B3g)	1.35	0.39
830.46	(B3g)	0.09	0.01	658.89	(B3g)	6.08	1.71
855.78	(Ag)	0.35	0.05	689.96	(B3g)	0.69	0.18
929.02	(Ag)	14.13	1.92	698.15	(B3g)	1.11	0.29
				698.62	(Ag)	19.28	5.03
				719.95	(B3g)	0.56	0.14
				742.07	(Ag)	15.52	3.75
				761.28	(B3g)	0.12	0.03
				765.11	(Ag)	21.07	4.89
				816.90	(Ag)	0.01	0
				829.08	(B3g)	0.08	0.02
				852.43	(Ag)	0.19	0.04
				927.29	(Ag)	9.61	1.75

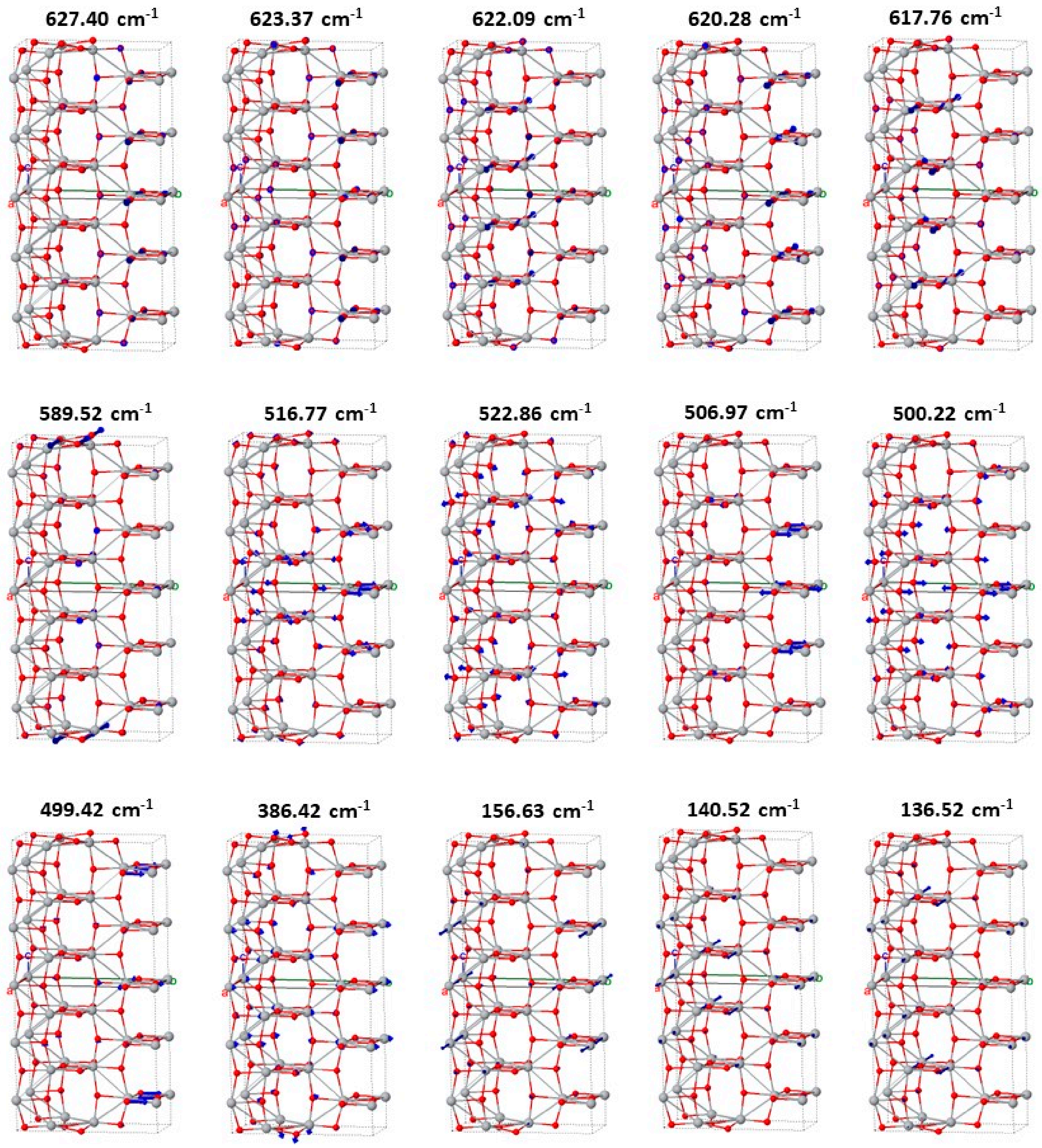

Figure S7. Visualization of the chosen modes for 11ML (100) structure. Figures were prepared using CRYSPLOT tool.

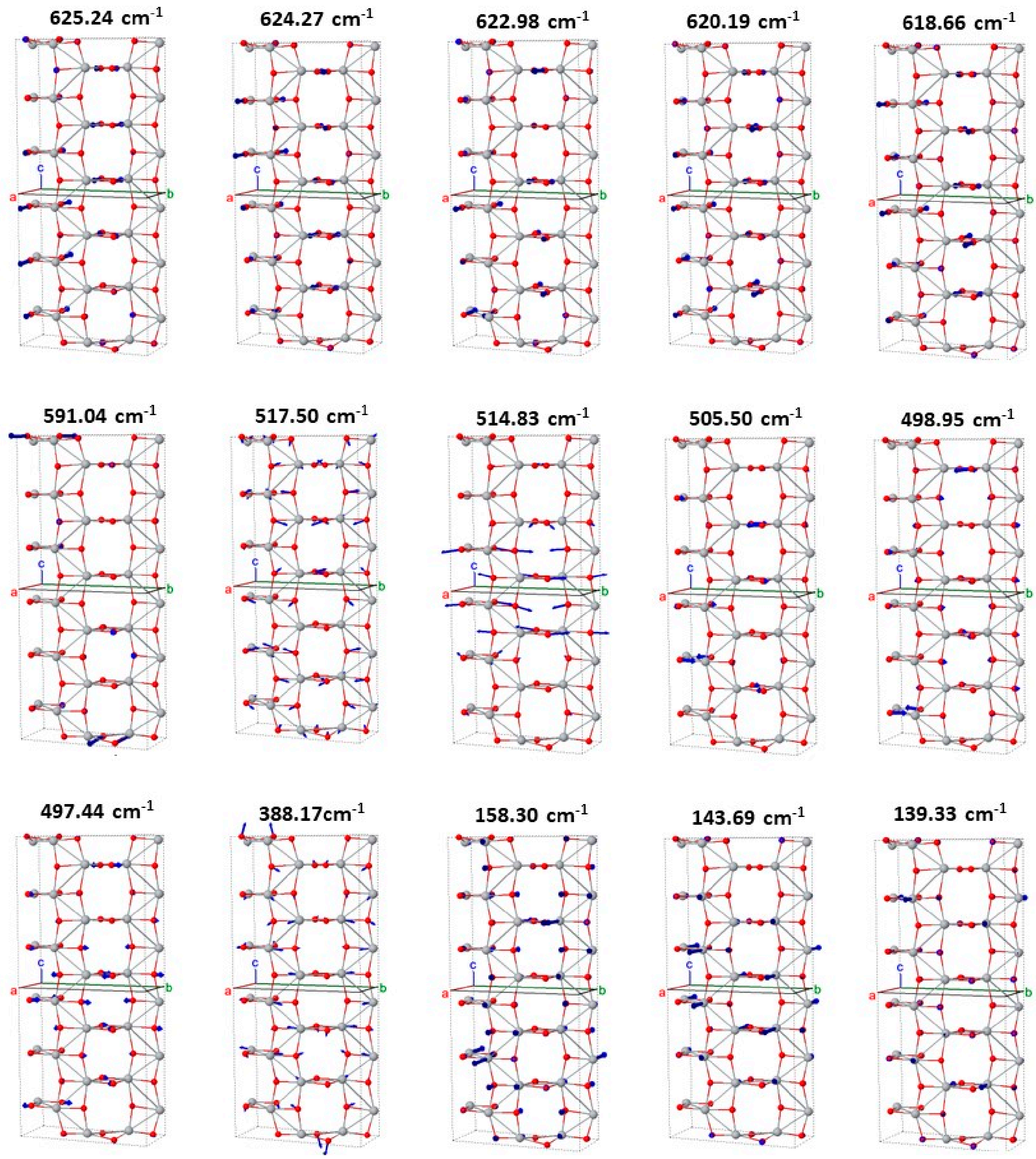

Figure S8. Visualization of the chosen modes for 12ML A(100) structure. Figures were prepared using CRYSPLOT tool.

Table S9. Frequencies and intensities (before temperature correction) for main “bulk-like” and “surface modes for **(101) termination.**

**Mode**	**6ML**		**8ML**		**10ML**		**12ML**		**14ML**		**18ML**		**20ML**	
	**Freq.**	**Intens.**	**Freq.**	**Intens.**	**Freq.**	**Intens.**	**Freq.**	**Intens.**	**Freq.**	**Intens.**	**Freq.**	**Intens.**	**Freq.**	**Intens.**
**"Eg(3)"**	622.64	899.46	623.61	1000.00	624.31	1000.00	624.77	1000.00	624.80	1000.00	625.31	1000.00	625.60	1000.00
					619.19	110.51	620.08	163.32	621.26	182.81	623.08	176.30	623.73	187.12
									618.34	34.45	620.08	67.69	621.07	66.42
											618.38	15.20	618.07	28.75
**"Surface"**	605.44	1000.00	603.20	661.86	602.12	519.85	601.87	422.60	601.87	366.44	602.65	279.84	603.16	253.03
**"A1g"**	513.66	414.14	511.86	329.11	516.32	216.14	515.58	235.05	517.63	2.27	517.04	199.60	518.91	94.17
									516.63	251.81	515.37	6.37	516.64	142.30
													515.44	19.69
**"B1g(2)"**	509.74	56.57	507.28	77.93	508.04	128.95	506.92	154.77	507.68	135.92	509.52	143.53	510.79	105.39
	502.67	33.15	505.14	93.06	505.72	2.12	504.63	89.36	505.65	3.64	506.57	2.31	505.78	37.77
					504.43	130.61	504.63	89.36	502.98	101.15	504.76	38.45	505.06	68.90
									501.49	4.34	502.45	44.19	503.90	34.11
											502.19	31.10	501.27	25.43
**"B1g(1)"**	429.22	305.18	413.59	187.54	406.72	234.53	402.31	65.72	409.84	58.02	409.57	39.53	416.91	33.46
	397.02	80.80	398.83	37.22	396.40	47.57	397.96	186.21	392.38	187.24	386.21	168.92	386.98	137.18
					385.79	22.50	381.94	28.28	389.01	42.42	387.86	78.58	385.83	129.00
**"Eg(1)"**	151.88	370.23	148.58	428.55	146.79	465.37	145.65	343.04	144.88	355.86	144.83	251.29	145.05	196.86
					145.55	6.95	144.52	154.74	144.03	142.32	144.14	152.18	144.44	90.41
									143.32	24.29	143.65	119.13	143.94	157.17
											143.40	19.42	143.15	106.71

Table S10. Frequencies and intensities (before temperature correction) for main “bulk-like” and “surface modes for **(001) termination (“symmetric”).**

**Mode**	**6ML**		**8ML**		**10ML**		**12ML**		**14ML**		**16ML**		**24ML**	
	**Freq.**	**Intens.**	**Freq.**	**Intens.**	**Freq.**	**Intens.**	**Freq.**	**Intens.**	**Freq.**	**Intens.**	**Freq.**	**Intens.**	**Freq.**	**Intens.**
"Eg(3)"	626.56	23.95	627.07	11.23	627.18	15.75	627.43	13.70	627.72	14.39	628.05	11.80	627.85	2.21
	624.31	12.37	625.48	21.85	625.12	27.27	626.26	2.47	626.21	6.21	626.76	1.75	627.57	4.44
			624.60	22.34	625.93	0.66	625.36	37.24	625.45	43.17	626.32	7.32	627.20	12.87
					624.85	31.81	625.70	0.59	626.46	0.00	625.66	43.72	626.70	27.79
							625.02	42.39	625.80	2.23	626.55	0.08	627.73	0.38
									625.31	51.15	626.87	0.21	627.43	7.03
											625.55	55.26	627.13	16.64
													626.66	27.66
"Surface"	528.80	1000.00	528.49	1000.00	528.59	1000.00	528.87	1000.00	529.22	1000.00	529.97	838.00	532.40	455.95
"A1g"	506.91	137.65	510.37	250.33	513.32	449.91	514.85	661.53	515.93	882.64	515.59	1000.00	518.03	1000.00
											510.07	29.05	515.37	20.66

													511.05	20.75
"B1g(2)"	494.23	87.57	499.99	193.83	501.66<br>496.65	209.89<br>77.11	504.88<br>499.38	136.90<br>240.28	507.31<br>501.61<br>496.19	51.09<br>370.54<br>41.94	502.93<br>500.11	270.84<br>167.88	505.89<br>502.92<br>500.75<br>497.84	101.52<br>290.22<br>14.01<br>1.59
"B1g(1)"	343.26	139.83	360.34	272.36	368.63	432.64	373.26	601.02	375.86	768.42	377.79	809.98	381.40	770.56
"Eg(1)"	146.66<br>149.71	9.58<br>6.53	145.57<br>145.88	13.45<br>9.66	145.09<br>145.20	17.31<br>13.84	144.94<br>144.85	20.86<br>17.67	144.75<br>144.82	23.70<br>21.36	145.33<br>145.08	24.20<br>21.91	146.62<br>146.04	18.54<br>15.65

Table S11. Frequencies and intensities (before temperature correction) for main "bulk-like" and "surface" modes for **(001) termination** (odd and even slabs are separated).

Mode	6ML		8ML		10ML		12ML		14ML	
	Freq.	Intens.	Freq.	Intens.	Freq.	Intens.	Freq.	Intens.	Freq.	Intens.
"Eg(3)"	625.88<br>622.57	1000.00<br>126.78	624.77<br>622.98<br>619.76	1000.00<br>149.11<br>52.87	624.50<br>623.53<br>620.74<br>618.97	1000.00<br>129.72<br>72.69<br>17.42	625.24<br>624.27<br>621.98<br>620.19<br>618.66	1000.00<br>138.73<br>181.54<br>17.11<br>18.34	625.94<br>624.32<br>622.93<br>621.07<br>620.05<br>617.93	1000.00<br>514.91<br>454.13<br>9.72<br>22.44<br>19.20
"Surface"	595.80	324.53	591.89	234.06	590.84	181.02	591.04	157.58	591.58	195.96
"A1g"	516.17	297.20	515.46	292.61	515.67	261.05	517.50<br>514.83	189.18<br>105.17	516.63<br>515.16	481.38<br>9.04
"B1g(2)"	496.57<br>485.54	134.32<br>19.60	503.84<br>498.73<br>492.19	5.50<br>165.89<br>5.84	504.21<br>500.21<br>497.78	19.15<br>140.32<br>4.39	505.50<br>498.95<br>497.50	18.30<br>182.63<br>13.09	504.63<br>501.61<br>500.12	10.22<br>163.90<br>62.70
"B1g(1)"	441.85	113.10	398.25	47.75	388.12	67.59	388.17	150.30	384.20	253.95
"Eg(1)"	148.67	217.61	144.56<br>154.78	320.18<br>11.52	143.65<br>140.11	308.46<br>62.27	143.69<br>139.33	360.95<br>72.85	143.97<br>141.73<br>138.96	500.52<br>110.37<br>70.07

Mode	5ML		7ML		9ML		11ML		13ML	
	Freq.	Intens.	Freq.	Intens.	Freq.	Intens.	Freq.	Intens.	Freq.	Intens.
"Eg(3)"	621.81<br>606.59	1000.00<br>512.79	623.98<br>619.04<br>616.69	1000.00<br>4.40<br>800.25	626.17<br>621.81<br>620.86<br>614.82	948.15<br>167.52<br>1000.00<br>67.13	627.40<br>623.37<br>622.09<br>620.28<br>617.76	588.05<br>210.65<br>1000.00<br>4.73<br>40.63	627.64<br>623.90<br>623.13<br>620.78<br>619.62<br>615.88	491.30<br>98.77<br>1000.00<br>7.99<br>37.65<br>7.19
"Surface"	577.39	482.85	584.82	395.93	588.05	351.17	589.52	233.16	590.67	171.78
"A1g"	548.23	142.09	523.42	237.00	523.81	25.17	522.86	59.27	518.88	116.08

					516.63	398.17	516.77	363.37	516.55	291.32
"B1g(2)"	497.91	410.70	504.47	14.12	502.28	6.41	506.97	30.11	504.74	3.31
	487.22	21.12	496.43	234.42	499.67	355.94	500.22	144.98	500.73	196.33
			487.79	123.15	491.04	31.46	499.24	79.96	498.20	11.10
"B1g(1)"	466.29	126.55	421.83	158.59	396.88	137.67	386.42	176.38	385.01	134.36
"Eg(1)"	121.07	168.19	129.65	333.82	136.89	492.33	140.29	462.28	142.46	417.15
					132.78	38.06	136.52	64.83	140.31	51.20
									137.82	52.66

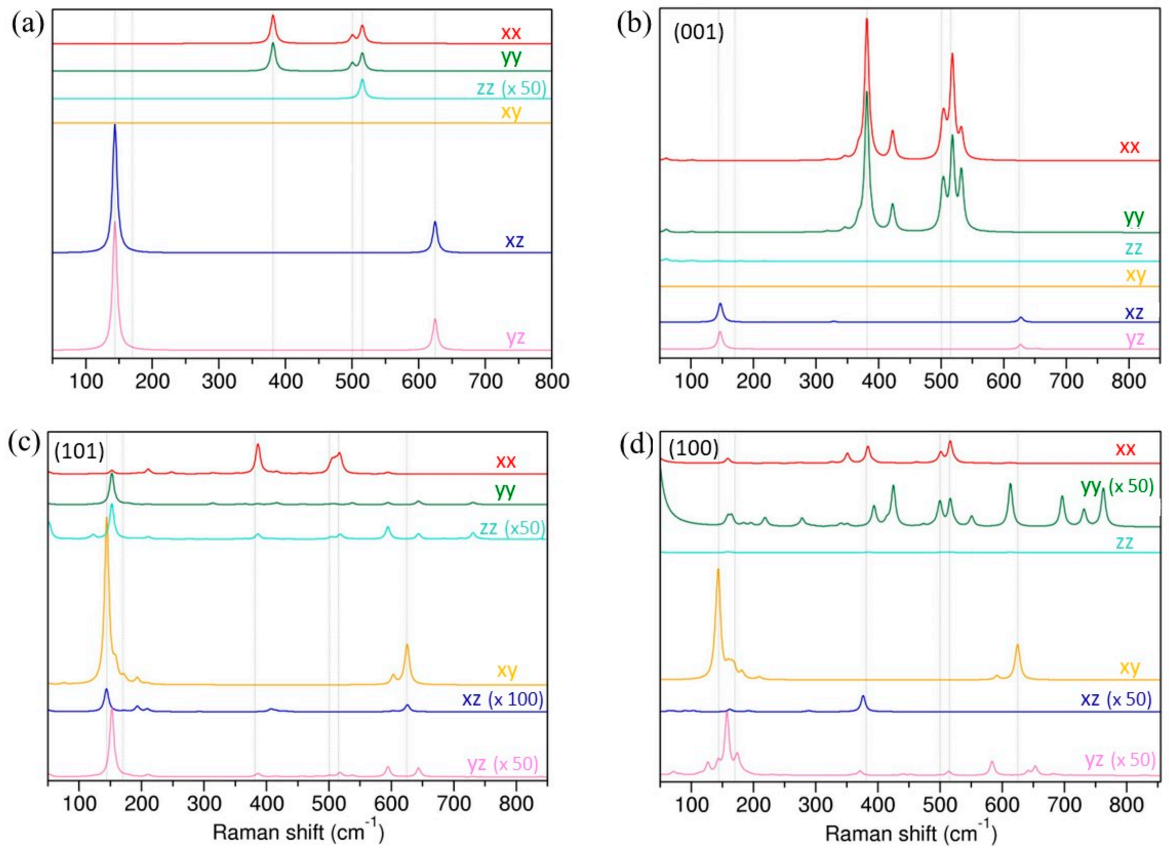

Figure S9. Single crystal Raman spectra for each inequivalent polarization direction for a) bulk anatase; b) (001) 24ML; c) (101) 20ML; d) (100) 14ML. Mind that in some cases the intensities were magnified to allowed their comparison (marked by "x" and the magnitude of enlargement in plots).
